# Chatbots to Support Mental Wellbeing of People Living in Rural Areas: Can User Groups Contribute to Co-design?

**DOI:** 10.1007/s41347-021-00222-6

**Published:** 2021-09-20

**Authors:** C. Potts, E. Ennis, R. B. Bond, M. D. Mulvenna, M. F. McTear, K. Boyd, T. Broderick, M. Malcolm, L. Kuosmanen, H. Nieminen, A. K. Vartiainen, C. Kostenius, B. Cahill, A. Vakaloudis, G. McConvey, S. O’Neill

**Affiliations:** 1 School of Computing, Ulster University, Newtownabbey, UK; 2 School of Psychology, Ulster University, Derry-Londonderry, UK; 3 School of Art, Ulster University, Belfast, UK; 4 Department of Sport, Leisure and Childhood Studies, Munster Technological University, Cork, Ireland; 5 NHS Western Isles, Stornoway, UK; 6 Department of Nursing Science, University of Eastern Finland, Kuopio, Finland; 7 Department of Health Sciences, Luleå University of Technology, Luleå, Sweden; 8 Nimbus Research Centre, Munster Technological University, Cork, Ireland; 9 Action Mental Health, Newtownards, UK

**Keywords:** Mental health, Co-creation, Conversational agents, Conversation design, Living labs

## Abstract

Digital technologies such as chatbots can be used in the field of mental health. In particular, chatbots can be used to support citizens living in sparsely populated areas who face problems such as poor access to mental health services, lack of 24/7 support, barriers to engagement, lack of age appropriate support and reductions in health budgets. The aim of this study was to establish if user groups can design content for a chatbot to support the mental wellbeing of individuals in rural areas. University students and staff, mental health professionals and mental health service users (*N* = 78 total) were recruited to workshops across Northern Ireland, Ireland, Scotland, Finland and Sweden. The findings revealed that participants wanted a positive chatbot that was able to listen, support, inform and build a rapport with users. Gamification could be used within the chatbot to increase user engagement and retention. Content within the chatbot could include validated mental health scales and appropriate response triggers, such as signposting to external resources should the user disclose potentially harmful information or suicidal intent. Overall, the workshop participants identified user needs which can be transformed into chatbot requirements. Responsible design of mental healthcare chatbots should consider what users want or need, but also what chatbot features artificial intelligence can competently facilitate and which features mental health professionals would endorse.

## Introduction

An emerging area of importance is the investigation of how digital technology can support rural mental health care (Benavides-Vaello et al., 2013). Chatbots, also known as conversational user interfaces, are a type of technology that can take diverse roles in supporting mental health. They are becoming increasingly popular as digital mental health and wellbeing interventions, with initial evaluations of efficacy showing promise (Hoermann et al., 2017; Provoost et al., 2017; Vaidyam et al., 2019). Chatbots may be geared towards a variety of outcomes such as medication adherence, treatment compliance, aftercare support, delivery of appointment reminders, user empowerment and improvement in the self-management of mental health and wellbeing through monitoring mood or symptom change (Hoermann et al., 2017). They can also be used to promote help-seeking (Hoermann et al., 2017). However, chatbots bring other potential benefits to supporting mental wellbeing which are widely recognised by practitioners and clients (Benavides-Vaello et al., 2013; Palanica et al., 2019; Provoost et al., 2017; Vaidyam et al., 2019). In addition to supporting those with mental ill health, digital technologies are also considered to have potential for preventing mental health problems and for improving the overall mental health of the population (Calvo et al., 2018). This is particularly relevant for those rural citizens living in social isolation who face compounded problems such as poor access to mental health services, no 24/7 support, barriers to engagement especially with older men, no age appropriate support, and reductions in health budgets (Benavides-Vaello et al., 2013). All of these factors further emphasize the need for resilience building services to avoid crisis interventions (Benavides-Vaello et al., 2013).

The evidence base is in the early stages and also product development requires improvement (Hoermann et al., 2017; Provoost et al., 2017; Vaidyam et al., 2019). Further research is necessary to determine how and if a digital technology intervention can be best used in the mental health sector and what developments or limitations need to be incorporated to make the intervention acceptable, effective and financially viable (Hoermann et al., 2017). Calvo et al. point out that the strength of digital technology may lie in the ability to provide an individual or personalised intervention and that traditional scales may not be the best way of measuring outcomes for digital interventions (Calvo et al., 2018). Queries include whether chatbots can move beyond interactions that are merely factually informative, and be able to incorporate emotional connotations either being overlooked or not understood (Morris et al., 2018). Conversational agents are limited in terms of their language comprehension abilities and emotional understanding which is a major source of user dissatisfaction (Morris et al., 2018). However, digital technologies are being used to support mental health with chatbots such as WoeBot and Wysa providing psychological assessment or the provision of psychoeducational materials (Fitzpatrick et al., 2017; Inkster et al., 2018). ‘Shim’ is another mental health chatbot previously designed for a non-clinical population to deliver cognitive behavioural therapy and strategies from positive psychology (Ly et al., 2017). There is an opportunity to increase access to a more meaningful style of symptom monitoring via a virtual “therapist” or “concerned friend” in the form of a chatbot. This means that such a technology would be natural, usable, and intuitive since it simulates everyday human-to-human conversation allowing the technology to be adopted by ‘non-digital’ natives. Further research is necessary to try to equip chatbots with an understanding of emotion-based conversation and appropriate empathic responses, to adjust their personality and mimic emotions (Morris et al., 2018). The question is whether or not machines will always be perceived as inferior to humans when it comes to emotions (Morris et al., 2018).

While many popular mental health chatbots exist, few studies have reported on how user groups can contribute to co-design as it is important to consider the user needs when designing content and features for this application. A few recent studies have involved young people in the design process to co-develop mental health and wellbeing chatbots targeted at under 18 s (Audrey et al., 2021; Grové, 2021). Another study by Easton et al. reported on co-designing content for a health chatbot by involving patients with lived experiences (Easton et al., 2019). However, to the best of our knowledge no study has reported on the involvement of stakeholders, which includes the general population, mental health professionals and service users in co-designing content for a mental health chatbot.

This study is part of a larger project called ‘ChatPal’, in which the objectives include the development and testing of a chatbot to support and promote mental wellbeing in rural areas across Europe. The overall aim of this study is to carry out workshops to establish if user groups can help to design a chatbot to promote good mental wellbeing in the general population, particularly for those living in sparsely populated areas. The objectives of the study are to:

(i) Gather general mental health wellbeing coping strategies recommended by workshop attendees
(ii) Gather and contrast views regarding the use of different scales for monitoring mental health, wellbeing and mood
(iii) Explore the range of personalities that chatbots can imbue and co-create chatbot personas preferred by the workshop attendees
(iv) Elicit the kind of questions asked by workers to clients in a mental health service (e.g. during a formal interaction) and enlist which questions would be suitable for a chatbot
(v) Co-create conversational scripts and user stories to inform dialogue and content design for a chatbot.

## Methods

Needs analysis workshops were carried out to gather the views of general population, mental health professionals and those with mental ill health. Workshops were based on the living labs methodology, with the idea that the design is not only user-centered but is also carried out by users (Dell’Era & Landoni, 2014). The living labs methodology offers advantages over other methods as it enables co-creation and engagement with service users and service providers primarily in the ideation and conceptualisation phases (Bond et al., 2015; Mulvenna & Martin, 2013); both stages of co-creation, focusing on the design of chatbot.

### Recruitment

Recruitment of participants varied based on region. In Northern Ireland, a recruitment email and participant information sheet were sent to students at Ulster University, inviting eligible individuals to attend. A similar approach was used at Action Mental Health (AMH) in Northern Ireland, with a recruitment email and participant information sheet sent to clients and additional recruitment posters put up on AMH premises. In Finland, university students, staff and mental health professionals were emailed invitations to attend the workshops. A snowballing technique, where study subjects recruit other acquaintances to participate, was used in Finland to recruit additional participants. In Scotland, mental healthcare professionals and service users were contacted via email and invited to attend. In Ireland, Cork University of Technology staff and students were contacted via email and invited to attend. In Sweden, welfare professionals working with young people were recruited by phone and e-mail.

For university staff and the general student population in Northern Ireland, Ireland and Scotland, the inclusion criteria was anyone over the age of 18; living in a rural area and with no history of a mental health diagnosis and no previous history of suicidal thoughts or behaviours in the past year. In Sweden, the inclusion criteria for welfare professionals included those working with supporting, aiding and/or treating young person’s mental wellbeing in the region of Norrbotten. In Finland, the inclusion criteria for university staff and students included anyone over the age of 18 and living in a rural area and for healthcare professionals included those over the age of 18; working in a rural region in the area of mental health and wellbeing. The requirements for mental health service users in Northern Ireland and Scotland included those who were users of the mental health/ mental wellbeing service at the time of the workshop; those with a history of mild-moderate anxiety and/or depression; and no suicidal thoughts or behaviours in the past year.

Due to the coronavirus pandemic, the workshops in Finland and Sweden took place virtually. All other workshops were face-to-face and took place prior to the pandemic.

### Workshop Details and Analysis

The schedule for the workshop involved a review of current mental health services, coping strategies, mental wellbeing scales, user story requirements, chatbot demo and persona development. The template for the workshops was designed by Ulster University and was structured as follows. At the beginning of the workshop, participants were provided with a single questionnaire to collect demographics and levels of digital health literacies. Participants were then split into small groups, with one rapporteur at each table to take notes and qualitative data. Each table was assigned a series of tasks or topics to discuss for approximately 15 minutes. A total of 10 topics/ tasks were discussed at each table.

1. *Mental wellbeing needs of people living in rural and sparsely populated areas* e.g. what affects quality of life for people with mental health difficulties? What are the things that make life good/bad for you?
2. *Pros and cons of current mental health services they may have used or know about.* How have mental health services or practitioners helped or hindered recovery? This was asked on a hypothetical basis for students and the general population with no mental health problems.
3. *Everyday coping strategies that participants believe support emotional resilience, higher moods and better overall mental wellbeing.* Discussion around medications, side effects, therapeutic benefits and leisure activities and other coping strategies.
4. *Analysis of short mental health survey scales regarding their fitness for purpose in regularly monitoring wellbeing.* Participants were presented with scales and discussed their utility for regularly monitoring wellbeing. The scales, which included Clinical Outcomes Routine Evaluation 10 (CORE-10) (Barkham et al., 2013), Patient Health Questionnaire-9 (PHQ-9) (Kroenke et al., 2001), and Warwick Edinburgh Mental Wellbeing Scale (WEMWBS) (Tennant et al., 2007) were chosen as they are commonly administered and could potentially be used by the chatbot. CORE-10 was validated in primary care patients for screening and review. It is easy to administer and is recommended for repeated use across therapy sessions, having a broad coverage, including depression and anxiety but also risk to self and general, social, and close relationship problems (Barkham et al., 2013). The PHQ-9 is a reliable measure of depression severity and response to treatment and it has been validated with a large sample of patients from primary care and obstetrics-gynecology clinics (Kroenke et al., 2001). WEMWBS was developed to monitor wellbeing, with a focus on positive aspects of mental health (Tennant et al., 2007). It has been validated for use in different locations, languages and cultures, and across many different settings for example in health services, workplaces and schools (Tennant et al., 2007). Discussions were around what is important in relation to the experience of mental illness, and what should be included in the scales.
5. *Demonstration of chatbot technologies and a mental health chatbot.* Videos were shown to participants including demonstrations of Amazon Alexa and Google Assistant as well as an overview video of WoeBot from the creators Youtube channel: ‘Meet WoeBot’. Participants then discussed the positive and negative aspects of chatbot technologies.
6. *Participants provided with hypothetical personalities that a chatbot can imbue and tasked to discuss these whilst providing their preferred persona of a chatbot.* Two example personas (Appendix I) were shared with participants. This allowed for discussions around what characteristics they would like within a chatbot and what role they feel the chatbot should take in terms of gender, personality traits etc. The participants were provided with a blank persona template (Appendix I) to help with designing the chatbot personality.
7. *Consideration of the kind of questions asked by workers to clients in a mental health service (e.g. during a formal interaction) and questions would be suitable for a chatbot.* Discussions focused around what would be important in conversations that a client and therapist might have.
8. *Co-designing chatbot dialogue.* Participants discussed how they might converse with a chatbot in general and whether or not they thought that it might be useful in monitoring their wellbeing. This was also discussed in relation to someone who was feeling mentally unwell.
9. *Mood monitoring.* Participants were asked how they would like a chatbot to monitor their moods. For example, using questions or emojis or allowing the chatbot to determine mood by analysing user text responses (sentiment analysis).
10. * Defining chatbot requirements or features.* This was done by collecting ‘user stories’ to inform the design of a chatbot. User stories are simply expressed descriptions of a chatbot feature as told from the perspective of a user or related stakeholder of the chatbot service. In the workshops, they were written as short sentences in the form “As a < type of user > , I want < some goal > because < some reason > .” These were written on post-it cards which were collected and shared on white boards for discussion. This was to enable the user-centred co-creation process to thrive.

This template was shared with partners in Ireland, Scotland, Finland, and Sweden so all workshops followed a similar structure, albeit some workshops took place virtually because of the COVID-19 pandemic restrictions on public meetings. Information gathered at each workshop was collated for the overall needs analysis results. Thematic analysis of user stories was conducted using an inductive approach to identify themes for chatbot design.

## Results

### Participants

A total of 78 participants were recruited to workshops across several European regions, including Northern Ireland (*N* = 21), Scotland (*N* = 14), Ireland (*N* = 24), Sweden (*N* = 5) and Finland (*N* = 14). Participants of the workshops included mental health service users (*N* = 11), university staff and students (*N* = 40) and mental health care professionals (*N* = 27). Participant demographic information was collected at workshops in Northern Ireland, Finland and Sweden (Table 1). This information was not available for workshop attendees in Scotland and Ireland.

**Table 1:** Participant information from workshops in Northern Ireland (NI), Finland (FIN) and Sweden (SWE)

	Workshop 1 (NI) *n* = 12	Workshop 2 (NI) *n* = 9	Workshop 4 (FIN) *n* = 4	Workshops 5 & 6 (FIN) *n* = 10	Workshop 7 (SWE) *n* = 5
Gender *n* (%)					
Male	3 (25%)	8 (89%)	0	0	0
Female	9 (75%)	1 (11%)	4 (100%)	10 (100%)	5 (100%)
Unknown	–	–	–	–	
Age					
Range	21–27	23–67	27–40	27–49	44–55
Mean (SD)	22.8 (1.8)	52.2 (10.5)	32.3 (5.9)	36.7 (8.2)	47.8 (3.9)
Computer literacy *n* (%)					
1 (Low)	–	1 (11%)	–	–	–
2	–	1 (11%)	–	1 (10%)	1 (20%)
3	2 (17%)	1 (11%)	2 (50%)	4 (40%)	3 (60%)
4	1 (8%)	2 (22%)	1 (25%)	2 (20%)	1 (20%)
5 (High)	2 (17%)	4 (45%)	1 (25%)	2 (20%)	–
Unknown	7 (58%)	–	–	1 (10%)	–
Has a smartphone *n* (%)					
Yes	6 (50%)	9 (100%)	4 (100%)	9 (90%)	5 (100%)
No	–	–	–	–	–
Unknown	6 (50%)	–	–	1 (10%)	–
Uses Facebook *n* (%)					
Yes	6 (50%)	5 (56%)	3 (75%)	7 (70%)	5 (100%)
No	–	–	1 (25%)	2 (20%)	–
Unknown	6 (50%)	4 (44%)	–	1 (10%)	–
Uses Twitter *n* (%)					
Yes	2 (17%)	2 (22%)	3 (75%)	1 (10%)	1 (20%)
No	4 (33%)	1 (11%)	1 (25%)	8 (80%)	4 (80%)
Unknown	6 (50%)	6 (67%)	–	1 (10%)	–
Uses snapchat *n* (%)					
Yes	5 (42%)	1 (11%)	–	1 (10%)	2 (40%)
No	1 (8%)	1 (11%)	4 (100%)	8 (80%)	3 (60%)
Unknown	6 (50%)	7 (78%)	–	1 (10%)	–
Uses google Alexa *n* (%)					
Yes	3 (25%)	3 (33%)	–	–	2 (40%)
No	3 (25%)	1 (11%)	4 (100%)	9 (90%)	3 (60%)
Unknown	6 (50%)	5 (56%)	–	1 (10%)	–

### Coping Strategies

Coping strategies were identified to support emotional resilience, positive mood and better overall mental wellbeing. Everyday coping strategies discussed in the workshops fell under the categories of spirituality, leisure, and others (Table 2).

**Table 2:** Coping strategies that support mental wellbeing

Spirituality	Leisure	Others
Meditation Listening to own thoughts and feelings Living according one’s own values Mindfulness (mindful encounters with others as well) Acceptive thinking, self-compassion Optimism, no rumination or worrying for the future Religion and prayer	Physical exercise Exploring nature Meaningful hobbies/activities Relaxing Listening to music Good recovering, i.e. sleep	Emotional skills Social relationships, friends, family Apps for mental wellbeing Balance in everyday life, routines, time for yourself Place/role where one can feel appreciated, accepted, and useful Basic knowledge about mental health Feeling of manageability and capability Wellbeing at work Feeling safe Financial safety Diet

### Mental Wellbeing Scales

Common mental health and wellbeing scales including CORE-10 (Barkham et al., 2013), PHQ-9 (Kroenke et al., 2001) and WEMWBS (Tennant et al., 2007) were shown to participants to identify positive and negative aspects and missing items which could help when it comes to choosing which scales to use in the chatbot. Overall, positive aspects that were discussed included that scales were short and to the point; useful to show changes over time if administered regularly; important for getting a general overview; useful starting point; able to help identify problems; and easy to understand. Negative aspects included that perhaps there were not enough questions to assess wellbeing; scales may be inaccurate or lead to a ‘false diagnosis’; certain questions could be triggers for person; regular use could affect answers; not personalised or too impersonal. Participants also felt that there were missing aspects to the scales presented, such as the lack of positive questions and questions specific to individual needs; options for multiple choice questions and tick box answers; lack of questions on emotions; missing questions around suicidal intentions.

### Chatbot Personas and Interactions

Participants were presented with video demonstrations on chatbot technology and shown examples of current popular mental health chatbots. This facilitated a discussion on the strengths and weaknesses of chatbot technologies (Table 3). Accessibility and functionality were identified as both positive and negative aspects. Availability, universality, functionality, and anonymity were discussed as benefits of a chatbot service (Table 3). Additional quotes from participants on the strengths of chatbots include:

Some people might open up to it more because it’s not human and they don’t feel judged. You can be more honest with it. This might be good for people who could do with face to face human support but aren’t quite ready for it—this might be the first step to speak to the chatbot.

It could help people who are working as well—because you can access quickly and easily—even for mental health workers! It’s interesting to think about workers because they can’t access services that are only open 9 to 5. This could be a way of complementing those services.

I suppose it would be easiest to access on the phone, its discrete, you can do it anywhere you can take it with you.

I can see a way of using it with our older service users… I can imagine a way of just… using it to talk—a way of having a conversation; just to talk to someone… I would have to have a lot more understanding of the mechanics of it and the type of conversation it might then be having with my older service users before I would recommend it or signpost them to it. You are gauging whether it’s right for someone… If it’s around social isolation—the man I saw last week is [over 90], lives alone, and doesn’t want to leave the house so just in terms of giving him some companionship or giving him something to talk about…

**Table 3:** Strengths and weaknesses of chatbots for mental wellbeing

Strengths	Weaknesses
Availability Available 24 hours a day, 7 days a week	Robotic intelligence Lacks intuition; not empathetic; may get easily confused (e.g. regional language/idioms)
Accessibility Easily accessible—especially for youth; instant replies; portable; good ‘first step in solving problems’	Accessibility Dependent on internet access
Universality ‘Same quality to everyone in all times’; no assumptions/personal history	Inflexibility Rigid, limited; discussion does not flow; user may need to repeat themselves
Functionality Remembers earlier discussions and combines things; points out strengths/positive things potentially unseen; reminders, and active online signposting	Functionality Could be difficult to pick up on mental health difficulties/suicidal ideation
Anonymity	Impersonal No personal history/assumptions; lacks body language and ability to read body language

Negative attributes identified by participants included robotic intelligence and inflexibility, some also felt they are impersonal (Table 3). Additional quotes from participants on the weaknesses of chatbots include:

I wouldn’t talk to the chatbot about things if I was having a very bad mental health day, I need a person. I would talk to it if I was having an ok day—it would depend how wobbly you are, how ok your day is.

It concerned me, what if someone is thinking about suicide or self-harm? What can this chatbot do to help? This is a very different situation to someone just saying ‘I fancy a chat about movies because I’m a bit lonely’. How does [the chatbot] pick up on suicidal ideation? At what point does it pick up on certain things? Can it tune in to if things aren’t right with a person? That worries me a bit.

**Fig. 1:**
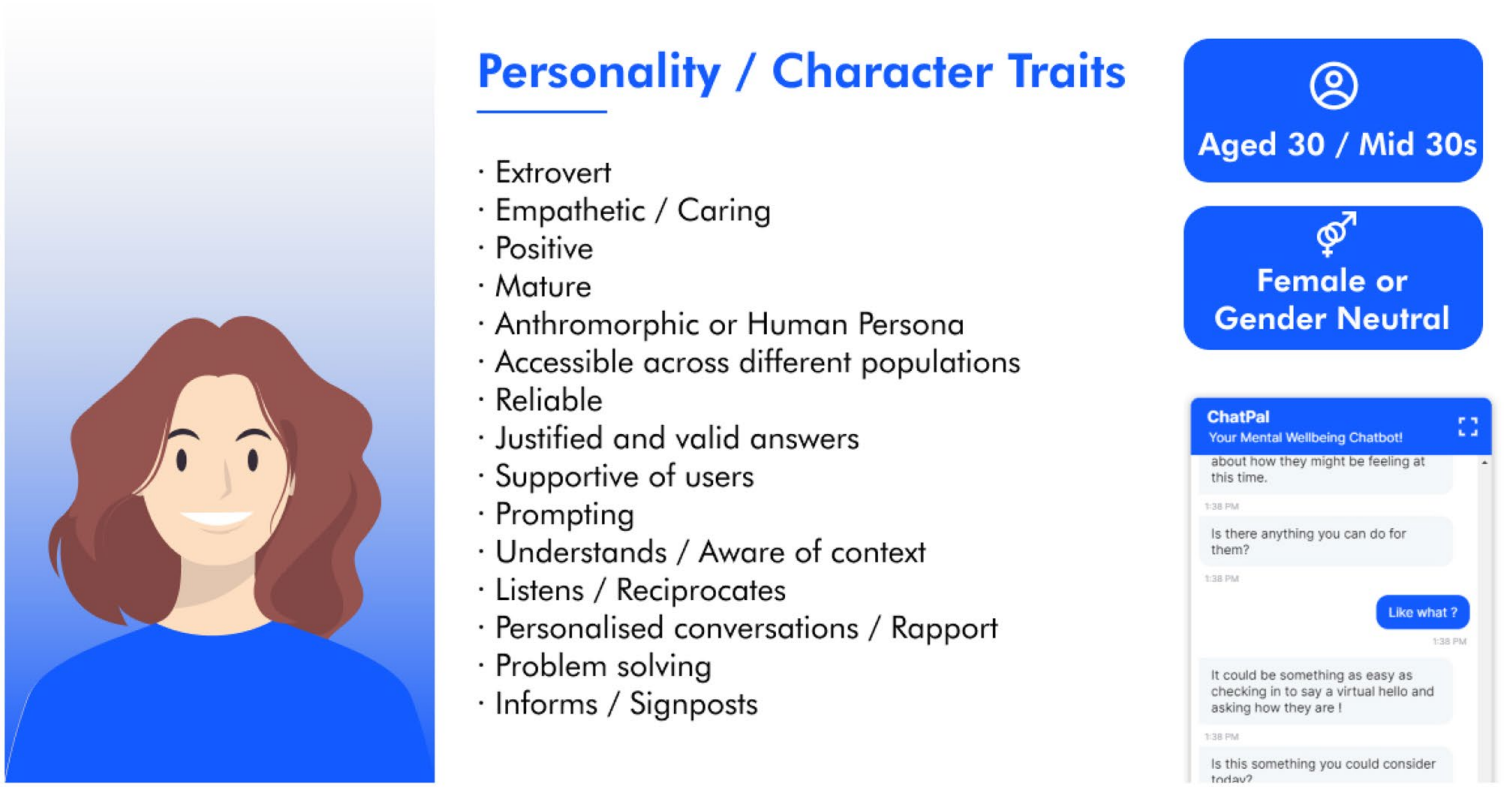
Desirable chatbot persona based on collated participant feedback

Each table was given hypothetical personalities that a chatbot can imbue and tasked with discussing the personas. Participants were asked to provide their preferred chatbot traits and qualities. The collated responses of participants were used to develop an overall chatbot persona with desired age, gender, personality, and character traits (Fig. 1). Overall, participants preferred the chatbot to be female or general neutral, aged around 30 years old (Fig. 1). The desired personality was a conversational agent that had a positive outlook, was widely accessible for different groups of people, and provided support to the user. Participants were keen to have a chatbot that was reliable, provided suitable answers and useful information but also one that also knows when to listen and prompt users. Participants also felt it was important to build a rapport with the chatbot so the interactions felt personal and that the chatbot could understand and be aware of the context of the conversation.

The types and examples of initial and follow-up interactions that individuals would like to have with a chatbot were discussed (Table 4).

**Table 4:** Types and examples of initial and follow-up interactions with the chatbot

Initial interactions with chatbot	Examples
Introductions/building rapport	Hi, would you like to tell me your name? How can I help? What do you want to talk about? What would you like to tell me today? Explain that everything they say is confidential
Assessment of emotional state (and discussing what would improve it)	How can I help you right now? What is on your mind How are you feeling today?

Follow-up interaction with chatbot	Examples
Asks something about topics discussed last time	Do you want to continue the conversation? Last time we discussed…how is this now? What kind of thoughts have you had since the last time we met?
Make user aware of additional support	Do you need further support? Is there anyone else you talked to about this?
Direct to local resources (e.g. support groups) that can help with specific problems	Do you need further support?
Discussion on feelings mood before and after chatbot conversations	How do you feel now (after our talk)?
Could recognise mood patterns and based on this contact more often if needed	Do you want to continue the conversation?

### User Stories

User stories were collected from participants, which are simply descriptions of a chatbot feature or requirement. These were collected in the form of short sentences, “As a < user type > , I want < some goal > because < some reason > . Based on the user stories, key themes were identified (Table 5) which can inform chatbot design by defining requirements or writing dialogues to fit these themes.

**Table 5:** Themes identified from user stories which can be used to inform chatbot design

Theme	User story examples
Check in facility	“As a single parent, I want the chatbot to ask how I’m doing regularly, so that I could be more capable mother in daily life” “As a mental health service user, I want a chatbot to check in with how I am feeling while I am waiting for face-to-face treatment options to become available, so I am not felt feeling alone and without any support while I am on the waiting list”
Positive encouragement	“As a friend, I want chatbots for helping others, giving advice, encouraging positive health and providing a support system for others” “As a student, I want the chatbot to remind me that everything will be okay, so that I can gain a sense of hope”
Place to share feelings and interests	“As a rugby player, I want to talk about hobbies that I am interested in, so it feels that I am talking to a person rather than a robot” “As an older person experiencing social isolation, I want a friendly chatbot to talk to about my interests so I can feel less lonely & I can feel some degree of companionship in my home”
Support/someone to talk to	“As someone who suffers from mental health difficulties, I want a friendly, empathetic and understanding chatbot that can talk to me as a person following my mental health, even if it is terrible on a certain day”
Activities, games, or things to do	“As a student, I want interactive games to engage in helping to solve problems, so that it will keep me engaged” “As a part-time worker, I would like options of things to do on my day off, as it can help with my work life balance” “As an unemployed person, I want the chatbot to give me tips on activities I could do, so I would not just be in home doing nothing”
Signposting or links to resources and services	“As a student, I want after-care numbers / counselling information, so that I know that there is help available elsewhere” “As a student, I want chatbots for signposting to services and mental health advice so I can access tips for coping” “As a mental health professional, I want something which empowers service users to support their own mental health but also knows when they need to be signposted to seek out support from a real person”
Tools/tips to manage life problems	“As a student, I could get tips from chatbot related to studies, so that studying does not get too distressing or heavy” “As a user, I want the chatbot to remind me to taking my own time in daily life, so that I could cope better with my daily life”
Mood or symptom tracking	“As a user, I would like my mood measured by asking me questions, so that I would then be able to ask for my mood level and history to enable me to manage life)” “As a person with depression and anxiety, I think being able to track my moods would be really helpfulso that I could have early intervention to stop my mood dipping even further”
Mental health information/psychoeducation	“As a working age user, I want the chatbot to give me concrete advice to handle anxiety and insomnia, so I could cope with daily life better” “As a person who is confused with his mind and body reactions, I want a chatbot that offers information and psychoeducation” “As a user with anxiety, I want tools to manage anxiety in different situations, so creating and maintaining social relationships becomes easier”
Mind/feelings management skills	“As a user feeling ashamed, I want a chatbot to help me relieve my feeling of shame” “As a person who has lost a near relative, I want a chatbot to help me to feel secure”
Mental health scales	“As a psychology student, I want scales (reliable and valid) to be used to measure wellbeing, sleep disturbance etc.”
Dealing with triggers around mental health/suicidal behaviour	“As a mental health worker, I want the chatbot to respond appropriately to triggers, so that person in severe distress or at risk of harm will be helped by a healthcare professional”

## Discussion

### Principal Findings

The aim of this work is to assess if a chatbot for mental wellbeing could be co-designed with user groups through workshops across several European countries. This study benefited from the inclusion of participants who were engaged in services for their mental illness as well as those who self-declared that they were not experiencing a mental illness. Both groups are important to consider as the former have experience of face-to-face services, whereas the latter may be potential users of the future. User needs were identified at the workshops, which included different coping strategies for promoting overall good mental wellbeing, which could be provided as suggestions to the user. Alternatively, the suggested coping strategies could be used as a basis for developing content. There was agreement around the inclusion of validated mental health scales within the chatbot. Participants noted things that they felt are missing from the scales, such as a lack of positive questions, but these missing aspects or questions could be presented to the user as part of the conversation. Collectively, a chatbot that personified a female or gender-neutral character in their thirties is preferred. Participants felt it is important that the chatbot has generally positive personality traits as well as the ability to understand and connect with the user. The initial conversations with the chatbot could seek to build a rapport with the user to establish trust. Participants liked the idea of the chatbot regularly checking-in with the user, asking questions about emotional state or mood and tracking this over time. For repeated use of the chatbot, participants felt that reflecting on previous conversations would be beneficial. Many thought that the chatbot should provide a space to share thoughts and feelings but also provide information. This could be mental health education or simply sharing helpful tips or tools that could be used in everyday life. User retention and engagement with digital technologies can be challenging, however, participants suggested including gamification within the app which could combat this problem. Finally, given the risk that conversational agents may not respond appropriately to potential crisis situations around mental health or suicidal intent, it was suggested that the chatbot should have keyword triggers that signpost to external resources.

### Link with Previous Work

Chatbots were discussed as a place to simply share feelings. This would align with the concept of expressive writing around negative emotional experiences, which has been shown to be potentially important in maintaining mental health (Sabo Mordechay et al., 2019). Practicing gratitude can improve overall positive behaviour and emotions (Armenta et al., 2017) and gratitude diaries have suggested benefits in several contexts including the management of suicidal crises (Ducasse et al., 2019), post discharge psychiatric inpatients (Suhr et al., 2017) and occupational stress management in health care professionals (Cheng et al., 2015). Chatbots may provide a useful platform for such interventions, and the view would be to build in means of allowing the individual to self-monitor their wellbeing.

In individuals who are mentally unwell, there is often what is referred to as ‘low perceived need’ (Mojtabai et al., 2011), which means the individual typically does not recognise the intensity of their own illness. If chatbots were able to monitor wellbeing in terms such as visual analogue scales or something as simple as saying to the individual that their scores are intensifying, this may assist in promoting self-awareness and early intervention. Xu et al. (Xu et al., 2018) provided a review of current interventions to seek help for mental health problems and concluded that some interventions show efficacy in promoting formal help seeking, but the evidence for changes in informal help seeking is limited. Given the difficulties associated with mental health care services, for example waiting lists and the distance that people may have to travel in rural areas, digital technologies could play a role in both providing help and promoting help-seeking, particularly in an informal context. Availability, anonymity, and accessibility were noted as potential advantages to chatbots. However, potential issues such as empathy, being impersonal or rigid and internet access were noted for consideration. These results further strengthen the need for government investment in the provision of broadband, particularly now in view of Covid-19, as it could facilitate equal access to mental health care support. Chatbots can provide an anonymous platform to discuss mental health, which could be helpful for those who struggle to open up. For example, a recent study reported that soldiers returning from combat redeployment were two to four times more likely to report mental ill health symptoms on an anonymous survey compared to a non-anonymous survey (Warner et al., 2011). In regards to empathy, a recent study looked at the effectiveness of an empathic chatbot on mood following experiences of social exclusion (Gennaro et al., 2020). The authors found that participants using the chatbot which would respond empathetically had a more positive mood than those using a chatbot where responses were simply just acknowledged (Gennaro et al., 2020). Further research is needed in this area, as the challenge of being able to express empathy within chatbots is well recognised.

Chatbot personality is an important design consideration, and the desired user persona for chatbots may depend on the domain. In a recent scoping review on mental health chatbots, 3 studies that Abd-Alrazaq et al. looked at found that users would like to personalise their own chatbot by choosing the gender and appearance (Abd-Alrazaq et al., 2021). Another recent paper reported that young people wanted a chatbot with a gender neutral name that was inspiring, charismatic, fun, friendly and had an empathic and humorous personality (Grové, 2021). In our study, desirable features included a human persona who was female or gender neutral, aged approximately mid-thirties with an extroverted and supportive personality. Individuals wanted a platform to share thoughts in which the chatbot just listened or understood, which isn’t surprising as individuals in distress often do not share their deepest thoughts with close family members or close friends. Individuals in suicidal crises often report feelings such as perceived burdensomeness and thwarted belongingness (O’Connor & Nock, 2014). In these states, they typically do not feel a connection to their usual support networks and perceive themselves as a source of burden, which hinders them from disclosing their mental distress. Indeed, this issue around disclosure of mental illness and mental distress is particularly prevalent among mental health professionals themselves (Tay et al., 2018).

The scales used in current clinical settings were described as capturing many critical elements of the experience of mental ill health, but many other elements were noted as missing. Potentially useful additions included the ability to individualise the interaction, to have a diary and to specifically ask about suicidal intent. Initially many feared that the discussion of suicidal ideation might encourage such behaviours, but the research consistently shows that it is important to ask this question in an open way with ‘Question, Persuade and Refer’ being a well acknowledged approach (Aldrich et al., 2018).

Participants identified several coping strategies which they felt could play a role in supporting emotional resilience. Chatbots may play a role in promoting the actual use of these coping strategies, many of which have an evidence base and are supported by leading bodies such as the World health Organisation (WHO) (World Health Organisation, 2019) and the National Institute of Clinical Excellence (NICE) (National Institute of Clinical Excellence, 2019). In their times of crisis, males in particular typically show maladaptive coping strategies (e.g. consumption of alcohol or drugs or social withdrawal) (Department of Health Northern Ireland, 2019; O’Neill et al., 2014) and seek psychological help less than women (Addis & Mahalik, 2003). Gender differences in coping behaviours are evident in the literature, and women have been found to utilise more coping strategies than males (Tamres et al., 2002). A mental health chatbot could potentially help with this, as males could be more likely to open up to a chatbot if they were reluctant to attend face-to-face services.

### Implications

**Fig. 2:**
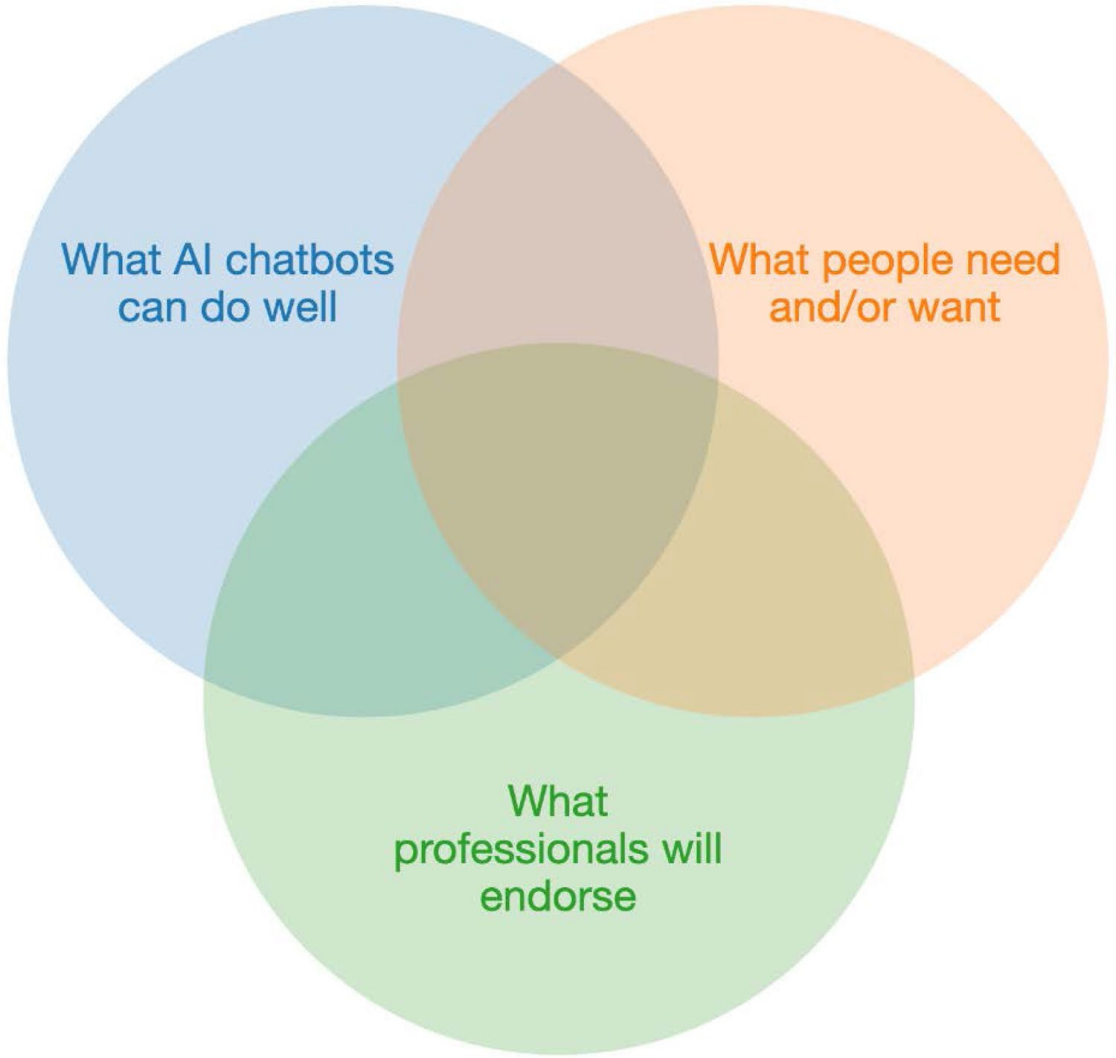
Stakeholder-centered approach for responsible mental health chatbot design

The results of the present study highlight what potential users of a mental wellbeing chatbot want or need. This is just one aspect to reflect on in relation to the design and development of mental health chatbots. It is crucial to look at approaches for responsible mental health chatbot design which could consider three things (1) what users say they need, (2) what chatbots and features mental health professionals would endorse, and (3) what AI chatbots can do well (Fig. 2). For example, chatbots can easily handle scripted dialogues with pre-defined replies or limited free text responses, and if users wanted a chatbot to self-diagnose or screen then it could be used to collect symptoms and use a decision flow to suggest a diagnosis. However, professionals may not be in support of this which could limit its credibility and widespread adoption. Alternatively, chatbots could be used for answering questions and signposting to paid mental health services, however, users may not want this type of application to direct to paid services and thus may avoid the technology altogether. Another example is a chatbot that supports free text, attempting to detect when a user is feeling depressed and tries to respond in a way that improves the persons mood. This may be endorsed by professionals but given the limitations of AI the responses may be inappropriate if the chatbot failed to understand what the user said or if it gave inappropriate advice. Therefore, a successful digital intervention could be thought of as the intersection between what users want and say they need, what professionals advocate and what AI does well as shown in Fig. 2.

### Limitations and Future Directions

In this study, people with previous suicidal thoughts and behaviours in the past year were not eligible to take part in the workshops. This is because we did not want any of the topics around mental health discussed in the workshops to cause distress to any participants. Nonetheless, we did include individuals with reported mental ill health as these are potential end users of this type of application.

The challenge now falls to disciplines such as computing and psychology to come together and advance the current provisions to match the features noted in the needs analysis. This is no easy feat as many practical and ethical issues need consideration. One of the main challenges with chatbot technologies in general lies with natural language processing (NLP), particularly in regards to free text (Kocaballi et al., 2020). Previous studies that have trialled mental health chatbots have reported issues with NLP including repetitiveness, shallowness and limitations in understanding and responding appropriately (Inkster et al., 2018; Ly et al., 2017). Another challenge is building technologies that are capable of competently responding to disclosures of intentions to harm the self or another. Previous work has looked at using machine learning approaches to detect suicidal ideation and self-harm from textual analysis of social media posts (Burnap et al., 2015; Roy et al., 2020). Future work could utilise similar methodologies in chatbots that are capable of competently responding to such disclosures. Other questions need to be addressed in the future. For example, How do we equip chatbots to respond to emotional statements, considering the wide array of human emotions and how these emotions are expressed? How do we provide follow-up care in a manner that matches the needs of the individual? To what extent is empathy necessary in the interaction or might the utility of chatbots lie primarily in providing the individual with a means to monitor their own wellbeing and any changes in it, and then signpost them to appropriate support services. This may be a very useful starting point given the well documented issues surrounding help seeking and service engagement.

### Conclusion

Overall, potential users recognise that chatbots may play a role in supporting mental health and they have clearly outlined their needs. In summary, user needs that can be used to inform chatbot design include: different coping strategies to promote good mental wellbeing; use of validated mental health scales; ask positive questions; provide educational content; reflect on previous conversations; elements of gamification; and keyword triggers to signpost to external resources. The desired persona was a female or gender neutral character, aged around 30, that could build a rapport and regularly check in with the user, allow them to track their mood and share thoughts. It is now important to transform these user needs into chatbot requirements whilst also considering which chatbot features AI can competently facilitate and which features mental health professionals would endorse. Future work must also consider the practical and ethical issues with chatbot technologies.

## Appendix

Appendix I: Example chatbot personas created for Woebot and Wysa, and blank template for completion by participants.
